# Phylogenetic conservation of the 3′ cryptic recombination signal sequence (3′cRSS) in the VH genes of jawed vertebrates

**DOI:** 10.3389/fimmu.2012.00392

**Published:** 2012-12-20

**Authors:** Yi Sun, Zhancai Liu, Zhaoyong Li, Zhengxing Lian, Yaofeng Zhao

**Affiliations:** ^1^State Key Laboratory of Agrobiotechnology, College of Biological Sciences, China Agricultural UniversityBeijing, China; ^2^Department of Biochemistry, Jiaozuo Teachers ColleagueJiaozuo, Henan, China; ^3^Beijing CRVAB Biotechnology Co., Ltd.Beijing, China; ^4^National Engineering Laboratory for Animal Breeding, Key Laboratory of Animal Genetics and Breeding of the Ministry of Agriculture, College of Animal Science and Technology, China Agricultural UniversityBeijing, China

**Keywords:** immunoglobulin, VH replacement, 3′cRSS, VH replacement footprint, charged amino acid, vertebrate

## Abstract

The VH replacement process is a RAG-mediated secondary recombination in which the variable region of a rearranged VHDJH is replaced by a different germline VH gene. In almost all human and mouse VH genes, two sequence features appear to be crucial for VH replacement. First, an embedded heptamer, which is located near the 3′ end of the rearranged VH gene, serves as a cryptic recombination signal sequence (3′cRSS) for the VH replacement process. Second, a short stretch of nucleotides located downstream of the 3′cRSS serve as a footprint of the original VH region, frequently encoding charged amino acids. In this review, we show that both of these two features are conserved in the VH genes of all jawed vertebrates, which suggests that the VH replacement process may be a conserved mechanism.

## Introduction

In vertebrates, the largely diversified repertoire in the variable domain of immunoglobulins is initially generated through V(D)J recombination in developing B lymphocytes. V(D)J recombination is a somatic, cell type-specific process in which the separated germline variable (V), diversity (D) (only for immunoglobulin heavy chain, IgH), and joining (J) gene segments are assembled to form an exon that encodes the functional variable domain. The V(D)J recombination process is also a site-specific recombination that depends on the recognition, binding, and cleavage of recombination signal sequences (RSSs) by the enzymes encoded by the recombination-activating genes 1 and 2 (*RAG-1* and *RAG-2*). RSSs flank all potentially functional V, D, and J gene segments. The consensus RSS consists of a conversed heptamer (CACAGTG) and an A-rich nonamer (ACAAAAACC), which are separated by approximately 12- or 23-bp spacers. V(D)J recombination occurs efficiently between two RSSs with different spacer lengths (Jung et al., 2006). Because the immunoglobulin gene loci of the IgH genes in tetrapods and teleosts are organized in a “translocon” fashion, a quasi-random selection and combination of the individual V, D, and J segments from the germline repertoire generates a large diversity of the antibody specificities (Flajnik, 2002; Nemazee, 2006). A further increase in this diversity is provided by the imprecise processing of the coding region junctions, including the deletion of nucleotides in the coding end and the addition of non-templated (N) nucleotides (Lieber et al., 2003).

Due to the stochastic nature of the V(D)J recombination process, B cell receptors (BCRs) that recognize autoantigens can trigger B cell central tolerance via anergy, clonal deletion, and receptor editing. Whereas anergy and clonal deletion inactivate or clear the self-reactive clones, receptor editing in immature B cells allows the cells to continue their immunoglobulin gene rearrangements to alter the specificity of their BCR (Nemazee, 2006). The secondary rearrangement occurs at the immunoglobulin kappa and lambda (Igκ and Igλ) loci, as previously demonstrated by many studies. However, the ongoing rearrangement of the IgH gene locus was once considered impossible because the primary rearrangement deletes the entire D locus and thus no D segments with the appropriate 5′ and 3′ 12-RSS sequences remain to join with new VH and JH segments (Gay et al., 1993; Tiegs et al., 1993; Prak and Weigert, 1995). Originally discovered in studies of murine pre-B cell lines, a type of secondary rearrangement called VH replacement, has now been documented as a receptor editing mechanism for the IgH gene in an increasing number of studies using knock-in mice and human normal and transformed B cells (Kleinfield et al., 1986; Reth et al., 1986; Chen et al., 1995; Cascalho et al., 1997; Zhang et al., 2003; Koralov et al., 2006). The VH replacement process closely resembles the mechanism of V(D)J recombination; this process uses a normal RSS of an upstream germline VH segment and a cryptic RSS (cRSS) that is embedded close to the 3′ end of the rearranged V exon to mediate a VH-to-VHDJH recombination. In addition, VH replacement usually produces junctional diversity or leads to frame-shifts *in vivo* (Covey et al., 1990; Chen et al., 1995; Koralov et al., 2006).

## Conservation of the 3′cRSS and a downstream charged amino acid-encoding nucleotide sequence in the VH genes of human and mouse

In almost all known human germline VH genes (47/51), the cRSS is composed of a heptamer (TACTGTG) in the opposite orientation to the RSS of the germline VH segments. In addition, no conserved nonamer similar to the consensus nonamer is located upstream of the heptamer (Covey et al., 1990; Radic and Zouali, 1996). Similar conserved heptamers have been identified in more than 60% of the mouse VH nucleotide sequences that are available in GenBank (Chen et al., 1995). Some studies suggested that the VH replacement process is a RAG-mediated recombination process because of the detection of the double-stranded DNA breaks at the cRSS and the extrachromosomal DNA circles. Zhang et al. provided further evidence that the recombinant RAG-1/RAG-2 proteins can cleave the cRSS *in vitro* (Covey et al., 1990; Usuda et al., 1992; Zhang et al., 2003). Furthermore, many additional 3′ cryptic recombination signal sequence (3′cRSS)-like motifs that only contain the most conserved trinucleotide of the heptamer, 5′CAC (or 3′GTG), in both orientations of the coding region of the VH gene have been considered to play a role in VH gene revision, which is a second receptor replacement mechanism that occurs in germinal center B cells that may have undergone clonal expansion in response to antigen stimulation (Itoh et al., 2000; Wilson et al., 2000). Some predicted cRSSs that are initiated by the CAC motifs have been found to support detectable levels of recombination in extrachromosomal recombination assays (Davila et al., 2007). Therefore, any heptamer that contains a CAC motif at its 5′ end may have the potential to act as a cRSS for secondary rearrangement.

During each round of VH replacement, the recipient VH may leave a short stretch of nucleotides downstream of the 3′cRSS as a footprint. The analysis of the VH replacement footprints (the residual 3′ sequences of the replaced VH at the V-D junctions) in natural human IgH sequences by Zhang et al. indicated that the footprints frequently contribute charged amino acids to the IgH CDR3 region, regardless of the reading frame. In addition, 80% of the amino acids encoded by the 3′ end of human VH genes in all three reading frames are highly charged (Zhang et al., 2003). In the mouse, the arginine (Arg)-encoding AGA codon was also found at the 3′ end of most VH genes (Koralov et al., 2006). Previous studies have indicated that somatic mutations to Arg are common in the majority of high-affinity anti-dsDNA antibodies generated in autoimmune mice (Radic et al., 1993). Because the germline D genes and the normal VH-D and D-JH junctions of the IgH gene in the human and mouse rarely encode charged amino acids, the antibodies that contain VH replacement footprints may have a tendency to become autoreactive (Zhang et al., 2004). In addition, antibodies containing an Arg-rich CDR3 are negatively selected in a mouse strain in which the IgH repertoire is generated by VH replacement, although the level of anti-DNA antibodies in the sera of these mutant mice is still elevated (Koralov et al., 2006). A similar observation was recently made in humans. In systematic lupus erythematosus (SLE) patients, the frequency of VH replacement is significantly higher than in healthy individuals, and more than half of the autoreactive antibodies are encoded by VH replacement products with CDR3 regions that are rich in charged amino acids (Fan, 2009).

The cRSS near the 3′ end of VH genes and the charged amino acid-encoding nucleotide sequence following the 3′cRSS are conserved in both human and mouse. However, the conservation of these two features is not comprehensive to all six groups of jawed vertebrates (cartilaginous fishes, teleosts, amphibians, reptiles, birds, and mammals). Because the genomic organization of the VH genes in cartilaginous fishes and birds does not provide an advantageous condition for VH replacement (McCormack et al., 1991; Dooley and Flajnik, 2006), we will present a detailed analysis of the VH genes in the other four classes of jawed vertebrates, including six mammals (mouse, Norway rat, guinea pig, rabbit, African elephant, and gray short-tailed opossum), two reptiles (painted turtle and anole lizard), one amphibian (western clawed frog), and three teleosts (zebrafish, Atlantic salmon, and channel catfish), to determine whether these two features have been conserved throughout the evolution of jawed vertebrates.

## Conservation of the 3′cRSS in the functional VH genes of different vertebrates

In our analysis, the functional germline VH sequences are available from the IMGT database (www.imgt.org) (for mouse and Norway rat), Ensembl genome database (www.ensembl.org) (for western clawed frog, painted turtle, and anole lizard) and other references (Ros et al., 2004; Danilova et al., 2005; Bengten et al., 2006; Wang et al., 2009; Yasuike et al., 2010; Guo et al., 2011, 2012).

Regarding only the canonical heptamer (TACTGTG) of the 3′cRSS, the percentage of VH genes with an embedded 3′cRSS varies widely among the listed species, from zero in the rabbit to 90.5% in the opossum (Table 1). If only those heptamers that contain the critical 3′ GTG (NNNNGTG) are considered to be functional 3′cRSSs, the 3′cRSS is present in more than 65% of the VH genes in all analyzed species except the channel catfish; in addition, the percentage of VH genes with this sequence is higher than 85% in most mammals (except the Norway rat), western clawed frog, zebrafish, and Atlantic salmon (Table 1). The first two nucleotides (GT) of the GTG motif of the 3′cRSS arise from the TGT codon for cysteine 104 (Cys104, IMGT numbering) and the third nucleotide (G) belonging to the following codon. More than 50% (67/122) of the heptamers that do not contain the GTG motif retain the GT nucleotides. In the majority of the germline VH genes from the most species analyzed below, the amino acid that follows Cys104 is alanine (Ala), which is encoded by a GCN codon (mouse 88/107, Norway rat 79/117, guinea pig 74/89, rabbit 12/12, African elephant 42/48, opossum 19/21, painted turtle 60/68, anole lizard 59/71, western clawed frog 31/38, zebrafish 32/33, and Atlantic salmon 43/50). Fanning et al. speculated that the 3′cRSS reflects the conservation of Cys104, which is critical for the structure of the H chain (Fanning et al., 1998). Our analysis supports this hypothesis, but the preference of the TGT codon for Cys104 and that of the following GCN codon for Ala is also important.

**Table 1:** **Frequency of the 3′cRSS heptamer in the functional germline VH genes of 12 species**.

**Species**	**VH family**	**Type of the heptamer at the 3′ end of VH genes**^**a**^			**References**
		**TACTGTG (CACTGTG) (%)**	**NNNNGTG (%)**	**Others (%)**	
**MAMMALS**					
Mouse^b^ *(Mus musculus)*	IGHV1 (J558)	56.9 (29/51)	94.1 (48/51)	5.9 (3/51)	IMGT database
	IGHV2 (Q52)	100 (8/8)	100 (8/8)	−	
	IGHV3 (36–60)	100 (6/6)	100 (6/6)	−	
	IGHV4 (X-24)	100 (1/1)	100 (1/1)	−	
	IGHV5 (7183)	90 (9/10)	90 (9/10)	10 (1/10)	
	IGHV6 (J606)	−	−	100 (5/5)	
	IGHV7 (S107)	100 (3/3)	100 (3/3)	−	
	IGHV8 (3609)	100 (6/6)	100 (6/6)	−	
	IGHV9 (VGAM3-8)	−	100 (4/4)	−	
	IGHV10 (VH10)	100 (2/2)	100 (2/2)	−	
	IGHV11 (CP3)	−	−	100 (2/2)	
	IGHV12 (CH27)	100 (1/1)	100 (1/1)	−	
	IGHV13 (3609N)	50 (1/2)	50 (1/2)	50 (1/2)	
	IGHV14 (SM7)	50 (2/4)	50 (2/4)	50 (2/4)	
	IGHV15 (VH15A)	100 (1/1)	100 (1/1)	−	
	IGHV16	−	100 (1/1)	−	
	**Total**	**64.5 (69/107)**	**86.9 (93/107)**	**13.1 (14/107)**	
Norway rat (*Rattus norvegicus*)	IGHV1	41.7 (10/24)	87.5 (21/24)	12.5 (3/24)	IMGT database
	IGHV2	28.1 (9/32)	59.4 (19/32)	40.6 (13/32)	
	IGHV3	100 (4/4)	100 (4/4)	−	
	IGHV4	50 (1/2)	50 (1/2)	50 (1/2)	
	IGHV5	71.4 (15/21)	80.9 (17/21)	19.1 (4/21)	
	IGHV6	12.5 (1/8)	12.5 (1/8)	87.5 (7/8)	
	IGHV7	66.7 (4/6)	66.7 (4/6)	33.3 (2/6)	
	IGHV8	87.5 (7/8)	87.5 (7/8)	12.5 (1/8)	
	IGHV9	−	75 (3/4)	25 (1/4)	
	IGHV10	−	−	100 (2/2)	
	IGHV11	40 (2/5)	40 (2/5)	60 (3/5)	
	IGHV12	−	−	100 (1/1)	
	**Total**	**45.3 (53/117)**	**67.5 (79/117)**	**32.5 (38/117)**	
Guinea pig (*Cavia porcellus*)	VH1	90.4 (19/21)	95.2 (20/21)	4.8 (1/21)	Guo et al., 2012
	VH2	93.8 (15/16)	100 (16/16)	−	
	VH3	78.8 (41/52)	84.6 (44/52)	15.4 (8/52)	
	**Total**	**84.3 (75/89)**	**89.9 (80/89)**	**10.1 (9/89)**	
Rabbit *(Oryctolagus cuniculus*)	IGHV1	−	100 (12/12)	−	Ros et al., 2004
	**Total**	−	**100 (12/12)**	−	
African elephant (*Loxodonta Africana*)	VH1	66.7 (2/3)	66.7 (2/3)	33.3 (1/3)	Guo et al., 2011
	VH2	100 (2/2)	100 (2/2)	−	
	VH3	83.3 (5/6)	83.3 (5/6)	16.7 (1/6)	
	VH4	85.3 (29/34)	88.2 (30/34)	11.8 (4/34)	
	VH5	100 (2/2)	100 (2/2)	−	
	VH7	100 (1/1)	100 (1/1)	−	
	**Total**	**85.4 (41/48)**	**87.5 (42/48)**	**12.5 (6/48)**	
Gray short-tailed opossum (*Monodelphis domestica*)	VH1	94.7 (18/19)	94.7 (18/19)	5.3 (1/19)	Wang et al., 2009
	VH2	−	100 (1/1)	−	
	VH3	100 (1/1)	100 (1/1)	−	
	**Total**	**90.5 (19/21)**	**95.2 (20/21)**	**4.8 (1/21)**	
**REPTILES**					
Painted turtle^c^ (*Chrysemys picta*)	VH1	89.7 (26/29)	96.6 (28/29)	3.4 (1/29)	Ensembl database
	VH2	10 (1/10)	10 (1/10)	90 (9/10)	
	VH3	100 (2/2)	100 (2/2)	−	
	VH4	−	−	100 (1/1)	
	VH5	100 (3/3)	100 (3/3)	−	
	VH6	−	−	100 (2/2)	
	VH7	50 (3/6)	50 (3/6)	50 (3/6)	
	VH8	100 (1/1)	100 (1/1)	−	
	VH9	75 (3/4)	75 (3/4)	25 (1/4)	
	VH10	100 (9/9)	100 (9/9)	−	
	VH11	−	100 (1/1)	−	
	**Total**	**70.6 (48/68)**	**75 (51/68)**	**25 (17/68)**	
Anole lizard^d^ (*Anolis carolinensis*)	Group I	46.7 (7/15)	86.7 (13/15)	13.3 (2/15)	Ensembl database
	Group II	35.3 (6/17)	76.5 (13/17)	23.5 (4/17)	
	Group III	53.3 (8/15)	80 (12/15)	20 (3/15)	
	Group IV	65 (13/20)	100 (20/20)	−	
	Ac VH38265	100 (1/1)	100 (1/1)	−	
	Ac VH338184	100 (1/1)	100 (1/1)	−	
	Ac VH377057	−	−	100 (1/1)	
	Ac VH405628	−	−	100 (1/1)	
	**Total**	**50.7 (36/71)**	**84.5 (60/71)**	**15.5 (11/71)**	
**AMPHIBIANS**					
Western clawed frog^e^ (*Xenopus tropicalis*)	VH1	90.9 (10/11)	100 (11/11)	−	Ensembl database
	VH2	83.3 (5/6)	83.3 (5/6)	16.7 (1/6)	
	VH3	−	20 (1/5)	80 (4/5)	
	VH4	50 (1/2)	100 (2/2)	−	
	VH5	100 (3/3)	100 (3/3)	−	
	VH6	100 (1/1)	100 (1/1)	−	
	VH8	100 (6/6)	100 (6/6)	−	
	VH9	−	100 (1/1)	−	
	VH10	100 (1/1)	100 (1/1)	−	
	VH11	100 (2/2)	100 (2/2)	−	
	**Total**	**76.3 (29/38)**	**86.8 (33/38)**	**13.2 (5/38)**	
**TELEOSTS**					
Zebrafish (*Danio rerio*)	IGHV1	100 (4/4)	100 (4/4)	−	Danilova et al., 2005
	IGHV2	100 (3/3)	100 (3/3)	−	
	IGHV3	−	100 (1/1)	−	
	IGHV4	85.7 (6/7)	85.7 (6/7)	14.3 (1/7)	
	IGHV5	−	66.7 (2/3)	33.3 (1/3)	
	IGHV6	100 (1/1)	100 (1/1)	−	
	IGHV7	100 (1/1)	100 (1/1)	−	
	IGHV8	75 (3/4)	100 (4/4)	−	
	IGHV9	50 (2/4)	50 (2/4)	50 (2/4)	
	IGHV10	−	100 (1/1)	−	
	IGHV11	100 (2/2)	100 (2/2)	−	
	IGHV13	−	100 (1/1)	−	
	IGHV14	100 (1/1)	100 (1/1)	−	
	**Total**	**69.7 (23/33)**	**87.9 (29/33)**	**12.1 (4/33)**	
Atlantic salmon (*Salmo salar*)	IGHV1	63.6 (7/11)	100 (11/11)	−	Yasuike et al., 2010
	IGHV2	100 (3/3)	100 (3/3)	−	
	IGHV3	−	100 (1/1)	−	
	IGHV4	−	80 (4/5)	20 (1/5)	
	IGHV6	12.5 (1/8)	100 (8/8)	−	
	IGHV7	100 (2/2)	100 (2/2)	−	
	IGHV8	100 (10/10)	100 (10/10)	−	
	IGHV9	−	100 (1/1)	−	
	IGHV10	100 (1/1)	100 (1/1)	−	
	IGHV12	100 (1/1)	100 (1/1)	−	
	IGHV15	100 (2/2)	100 (2/2)	−	
	IGHV16	100 (4/4)	100 (4/4)	−	
	IGHV17	−	100 (1/1)	−	
	**Total**	**62 (31/50)**	**98 (49/50)**	**2 (1/50)**	
Channel catfish (*Ictalurus punctatus*)	VH1	100 (2/2)	100 (2/2)	−	Bengten et al., 2006
	VH2	−	−	100 (1/1)	
	VH3	20 (1/5)	20 (1/5)	80 (4/5)	
	VH5	−	−	100 (1/1)	
	VH6	−	−	100 (2/2)	
	VH7	−	−	100 (5/5)	
	VH9	100 (1/1)	100 (1/1)	−	
	VH11	−	50 (1/2)	50 (1/2)	
	VH12	−	−	100 (2/2)	
	VH14	100 (1/1)	100 (1/1)	−	
	**Total**	**22.7 (5/22)**	**27.3 (6/22)**	**72.7 (16/22)**	

a The denominator inside the parentheses represents the total number of functional VH genes in the corresponding VH family, and the numerator represents the number of functional VH genes that contain the specified type of heptamer in the corresponding VH family. If a VH gene had more than one allele, only the 01 allele was used in the analysis of the 3′cRSS.

b All of the functional VH genes were obtained from the VH repertoire of the C57BL/6 strain (imgt.org/IMGTrepertoire).

c The functional germline VH genes of the painted turtle were identified from the genome scaffolds JH585110, JH585065, and JH585278 (pre.ensembl.org/Chrysemys_picta_bellii).

d The functional germline VH genes of the anole lizard were identified from the genome scaffold GL343491 (ensembl.org/Anolis_carolinensis). The groups of the anole lizard VH genes are classified according to the phylogenetic study by Gambon Deza et al. (2009). Ac VH338184, Ac VH377057, and Ac VH405628 are three functional germline VH genes that are not included in the following phylogenetic analysis.

e The functional germline VH genes of the western clawed frog were identified from the genome scaffolds GL173803, GL173909, and GL173564 (ensembl.org/Xenopus_tropicalis). Two VH genes from VH4 family are not included in the following phylogenetic analysis.

**Figure 1:**
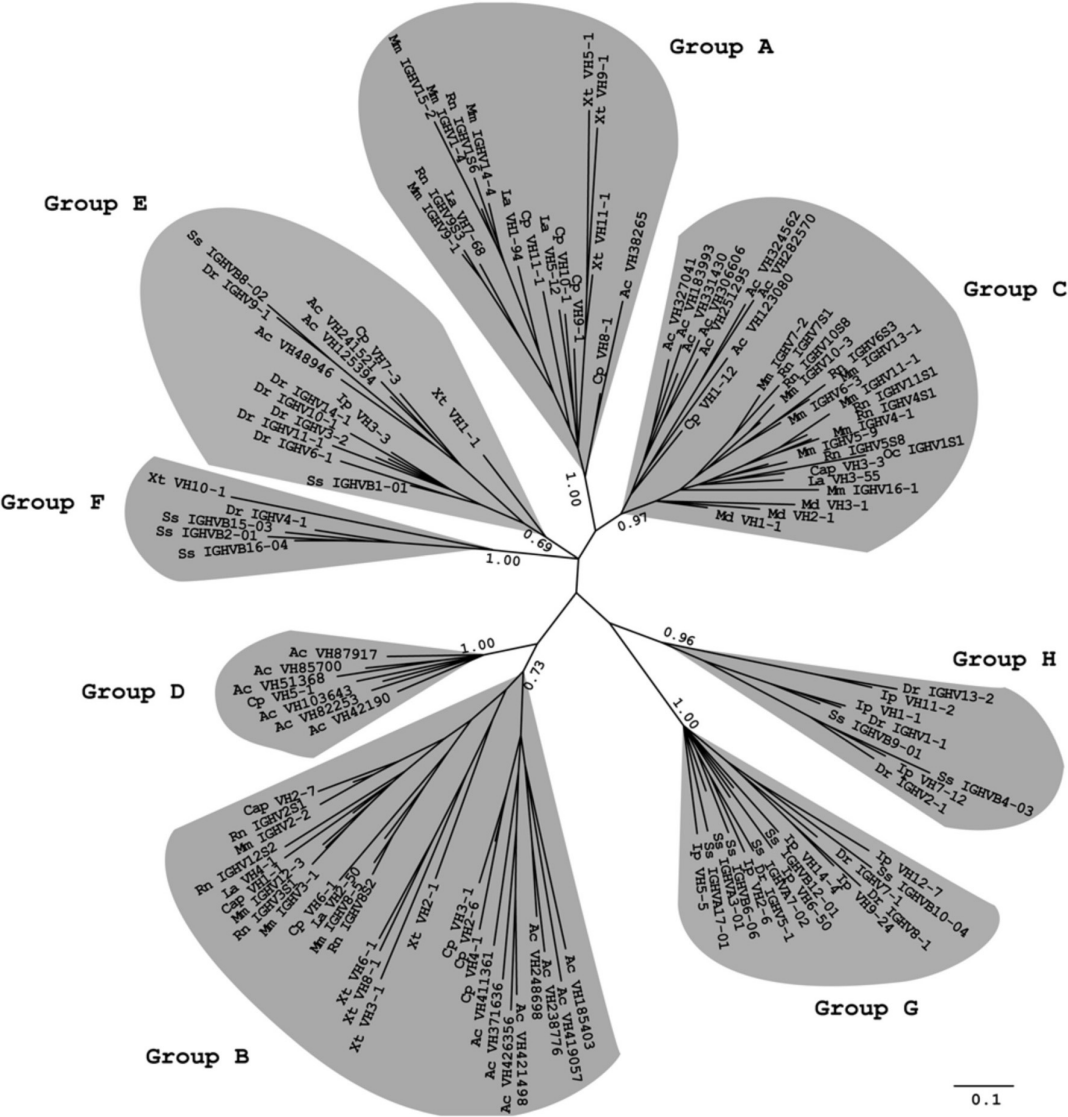
**Phylogenetic analysis of the VH genes from 12 species.** Phylogenetic trees were constructed using MrBayes3.1.2 and viewed in TreeView. Multiple DNA sequence alignments for the tree construction were performed using *ClustalX2*. Only the FR1-3 regions (as defined by the IMGT numbering system) of each sequence were utilized to construct the trees. Each VH family is represented with one sequence per species, which was chosen among the functional VH genes of the species. The optional VH genes of most species are named according to the nomenclature from the IMGT database (imgt.cines.fr) or other references. The only exceptions were the anole lizard, painted turtle, and western clawed frog, for which the VH genes were named using our own annotation. The species abbreviations used are as follows: Mm, mouse (*Mus musculus*); Rn, Norway rat (*Rattus norvegicus*); Cap, guinea pig (*Cavia porcellus*); Oc, rabbit (*Oryctolagus cuniculus*); La, African elephant (*Loxodonta Africana*); Md, gray short-tailed opossum (*Monodelphis domestica*); Cp, painted turtle (*Chrysemys picta*); Ac, anole lizard (*Anolis carolinensis*); Xt, western clawed frog (*Xenopus tropicalis*); Dr, zebrafish (*Danio rerio*); Ss, Atlantic salmon (*Salmo salar*); Ip, channel catfish (*Ictalurus punctatus*).

To further determine whether the maintenance of the 3′cRSS is driven by an evolutionary force, we used a phylogenetic analysis to classify the VH sequences from all 12 species into eight groups. The A, B, and C groups include the mammalian clans I, II, and III, respectively, as well as a few of the VH genes from painted turtle, anole lizard, and western clawed frog. Group D only contains the reptile VH genes. Group E consists of the VH genes from painted turtle, anole lizard, western clawed frog, and teleosts; group F contains VH genes from western clawed frog and teleosts. All VH genes from group G and H belong to the teleosts (Figure 1). We then calculated the frequency of the 3′cRSS in each VH group. As shown in Table 2, group A to F possess a high proportion of VH genes that contain the canonical heptamer motif (TACTGTG > 55%). By contrast, most VH genes from two teleost-specific VH groups, G and H, do not contain the canonical heptamer motifs (TACTGTG < 40%). However, all eight groups, regardless of the divergence time, contain high the proportion of VH genes that contain the critical 3′ GTG in the heptamer motif (NNNNGTG > 69%). Because the VH genes are subjected to divergent evolution by the birth-and-death process (Ota and Nei, 1994) and six VH groups (A–F) contain genes that have persisted for a long time in living species from different classes, the high frequency of the 3′cRSS in VH groups that evolved relatively later suggests that the maintenance of the 3′cRSS was positively selected during the evolution of the VH genes.

**Table 2:** **Frequency of the 3′cRSS heptamer in the functional germline VH genes of the eight groups**.

**Group**	**VH family**	**Type of the 3′cRSS heptamer**	
		**TACTGTG (CACTGTG) (%)**	**NNNNGTG (%)**
Group A	*Mus musculus* IGHV1 (J558), IGHV9 (VGAM3-8), IGHV14 (SM7), IGHV15 (VH15A)	56.9 (66/116)	90.5 (105/116)
	*Rattus norvegicus* IGHV1, IGHV9
	*Loxodonta africana* VH1, VH5, VH7
	*Chrysemys picta* VH8, VH9, VH10,VH11
	*Anolis carolinensis* Ac VH38265
	*Xenopus tropicalis* VH5, VH9, VH11
Group B	*Mus musculus* IGHV2 (Q52), IGHV3 (36–60), IGHV8 (3609), IGHV12 (CH27)	67.2 (127/189)	78.3 (148/189)
	*Rattus norvegicus* IGHV2, IGHV3, IGHV8, IGHV12
	*Cavia porcellus* VH1, VH2
	*Loxodonta Africana* VH2, VH4
	*Chrysemys picta* VH2, VH3, VH4, VH6
	*Anolis carolinensis* Group II
	*Xenopus tropicalis* VH2, VH3, VH6, VH8
Group C	*Mus musculus* IGHV4 (X-24), IGHV5 (7183), IGHV6 (J606), IGHV7 (S107), IGHV10 (VH10), IGHV11 (CP3), IGHV13 (3609N), IGHV16	67.3 (138/205)	79.5 (163/205)
	*Rattus norvegicus* IGHV4, IGHV5, IGHV6, IGHV7, IGHV10, IGHV11
	*Cavia porcellus* VH3
	*Oryctolagus cuniculus* IGHV1
	*Loxodonta Africana* VH3
	*Monodelphis domestica* VH1, VH2, VH3
	*Chrysemys picta* VH1
	*Anolis carolinensis* Group III
Group D	*Chrysemys picta* VH5	55.6 (10/18)	88.9 (16/18)
	*Anolis carolinensis* Group I
Group E	*Chrysemys picta* VH7	68.5 (50/73)	87.7 (64/73)
	*Anolis carolinensis* Group IV
	*Xenopus tropicalis* VH1
	*Danio rerio* IGHV3, IGHV6, IGHV9, IGHV10, IGHV11, IGHV14
	*Salmo salar* IGHV1, IGHV8
	*Ictalurus punctatus* VH3
Group F	*Xenopus tropicalis* VH10	94.1 (16/17)	94.1 (16/17)
	*Danio rerio* IGHV4
	*Salmo salar* IGHV2, IGHV15, IGHV16
Group G	*Danio rerio* IGHV5, IGHV7, IGHV8	36.7 (11/30)	76.7 (23/30)
	*Salmo salar* IGHV3, IGHV6, IGHV7, IGHV10, IGHV12, IGHV17
	*Ictalurus punctatus* VH2, VH5, VH6, VH9, VH12, VH14
Group H	*Danio rerio* IGHV1, IGHV2, IGHV13	39.1 (9/23)	69.6 (16/23)
	*Salmo salar* IGHV4, IGHV9
	*Ictalurus punctatus* VH1, VH7, VH11

## Conservation of the downstream charged amino acid-encoding nucleotide sequence in the functional VH genes of different vertebrates

**Figure 2:**
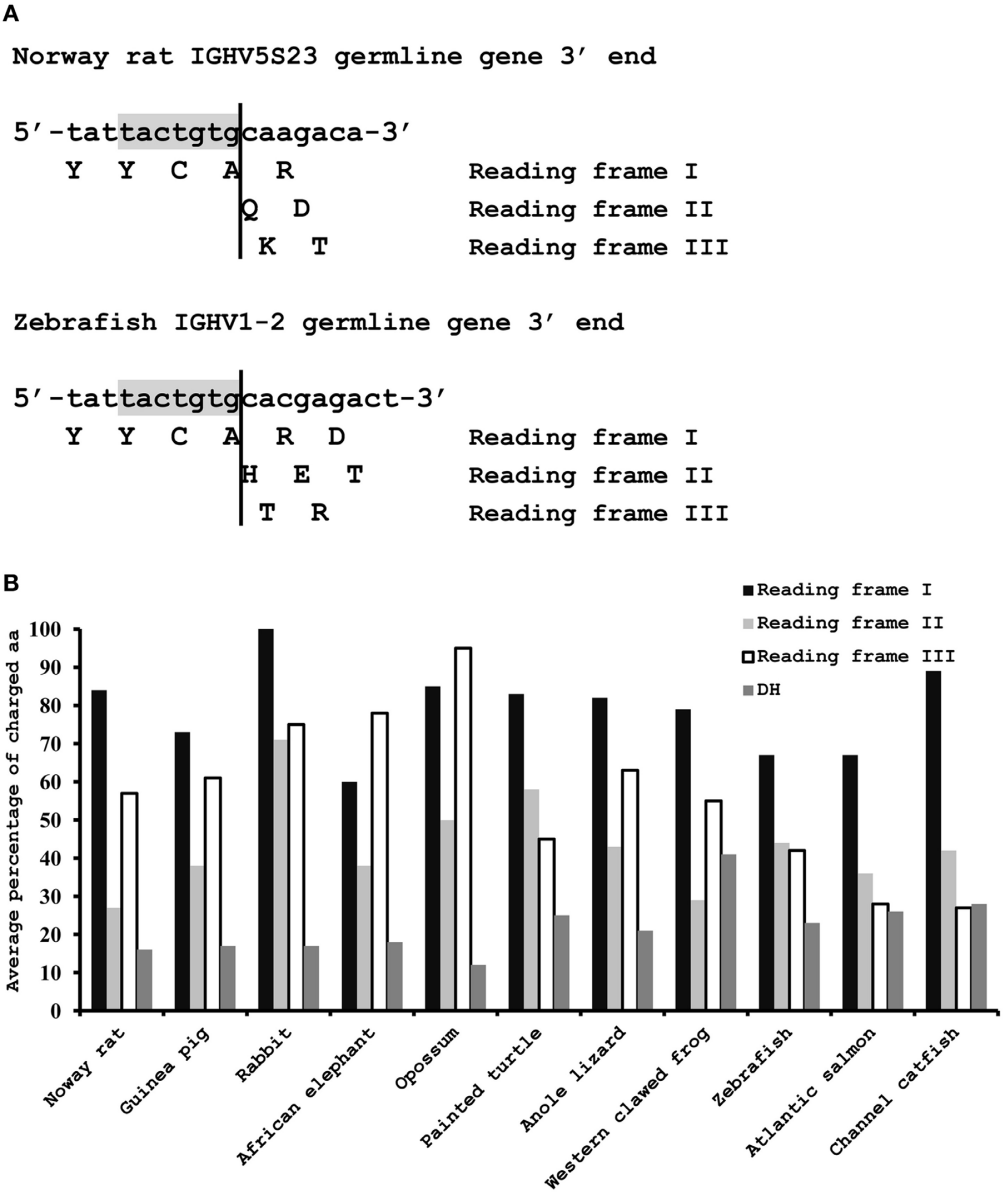
**Charged amino acids encoded by the nucleotide sequence following the 3′cRSS of the functional germline VH genes. (A)** Two examples of amino acids that are encoded by the nucleotide sequence following the 3′cRSS of the functional germline VH genes in three reading frames. The typical length of the nucleotide sequence following the 3′cRSS is 7 nt in tetrapods (above) and 9 nt in teleosts (below). The 3′cRSSs in the two sequences are shown in gray. **(B)** Average frequency of the charged amino acids encoded by the nucleotide sequence following the 3′cRSS of the functional germline VH genes in three reading frames and by all functional DH germline genes in 11 species. All functional germline DH sequences are available from the IMGT database (for Norway rat), Ensembl genome database (for painted turtle, genome scaffold JH584564), and other references (Ros et al., 2004; Danilova et al., 2005; Bengten et al., 2006; Zhao et al., 2006; Wang et al., 2009; Wei et al., 2009; Yasuike et al., 2010; Guo et al., 2011, 2012). For each DH gene, the reading frame which encodes the highest percentage of the charged amino acids are chosen to calculate the average percentage of the charged amino acids encoded by DH genes in certain species; thus, the actual average percentage of the charged amino acids encoded by the DH genes in certain species is much lower than the value showed in the figure.

The germline VH genes from 11 species were analyzed to determine the frequency of charged amino acids that are encoded by the nucleotide sequence following the 3′cRSS in three reading frames (Figure 2). The length of the nucleotide sequence following the 3′cRSS is usually 7 nt in seven tetrapod species and 9 nt in teleosts. Therefore, the number of amino acids that are encoded by this sequence in three reading frames is usually one, two, and two in tetrapods and two, three, and two in teleosts (Figure 2A). Due to the high percentage of A and G nucleotides in this sequence in all 11 species, the frequency of charged amino acids that are encoded by all three reading frames is higher than the random frequency (14/64, ~22%) and the frequency of the charged amino acids encoded by functional germline DH genes (Figure 2B). In addition, it is noteworthy that the frequency of charged amino acids encoded by reading frame I is greater than 60% for all 11 species (Figure 2B). Moreover, most of the charged amino acids are positively charged (Norway rat 108/115, guinea pig 65/69, rabbit 12/12, African elephant 28/30, opossum 17/17, painted turtle 65/70, anole lizard 71/81, western clawed frog 28/34, zebrafish 35/45, Atlantic salmon 45/63, and channel catfish 27/38). The reading frame I is the only correct reading frame that can ensure the encoding of a natural H chain protein; reading frame II and III might also be found in VH replacement footprints if the primary rearrangement is non-functional; thus, the VH replacement process in all 11 species should be prone to generate CDR3s that are rich in positively charged amino acids if this mechanism is conserved in jawed vertebrates.

## Conclusion

The main conclusion from the present analysis is that both the 3′cRSS and the charged amino acid-encoding nucleotide sequence following the 3′cRSS are conserved among different classes of vertebrates, which suggests that the VH replacement may be a conserved mechanism in all jawed vertebrates. However, additional experimental evidence from species other than human and mouse are needed to support this hypothesis. The biological function of the VH replacement process in non-mammalian vertebrates is worth careful and thorough study.

### Conflict of interest statement

The authors declare that the research was conducted in the absence of any commercial or financial relationships that could be construed as a potential conflict of interest.
